# One-lung Ventilation for Neonatal Pneumonectomy in a Hydropic Premature Infant: Fiberoptic-guided Left Mainstem Intubation

**DOI:** 10.4274/TJAR.2026.262718

**Published:** 2026-10-30

**Authors:** Zeliha Bozkurt, Büşra G. Erol, Nezir Yılmaz

**Affiliations:** 1 Adıyaman University Faculty of Medicine, Department of Anaesthesiology and Reanimation, Adıyaman, Türkiye; 2 Adıyaman Training and Research Hospital, Clinic of Anaesthesiology and Reanimation, Adıyaman, Türkiye

**Keywords:** One-lung ventilation, hydrops fetalis, neonatal anaesthesia, fiberoptic bronchoscopy, left mainstem intubation

## Abstract

Congenital cystic adenomatoid malformation (CCAM) is a rare lung anomaly that can cause severe respiratory distress and hydrops fetalis. One-lung ventilation (OLV) in neonates poses extreme anatomical challenges due to the lack of appropriately sized double-lumen tubes or bronchial blockers. A male neonate (2500 g, 30 weeks’ gestation) presented with hydrops fetalis and a massive right-sided thoracic CCAM. Due to progressive respiratory failure and hemodynamic instability, an emergency right pneumonectomy was scheduled on postnatal day 3. To achieve OLV, a 3.5-mm cuffed endotracheal tube (with the cuff left deflated) was advanced into the left main bronchus under fiberoptic bronchoscopy (FOB) guidance. Removal of the tube adaptor facilitated rapid, pragmatic placement while minimizing interruptions to ventilation during apneic oxygenation. Pressure-controlled lung-protective ventilation was maintained. Transient desaturation episodes during mediastinal manipulation were successfully managed. The 3-hour procedure was completed without intraoperative complications. In premature neonates in whom commercial lung-isolation devices are impractical, FOB-guided selective mainstem bronchial intubation is a rapid, feasible, and effective strategy for OLV. Individualized airway planning and FOB guidance are instrumental in ensuring accurate placement and maintaining oxygenation.

**Main Points**

• In low-birth-weight premature neonates in whom commercial bronchial blockers or double-lumen tubes cannot be deployed, selective mainstem bronchial intubation is a highly effective and definitive rescue strategy for lung isolation.

• Modifying the selective intubation technique by removing the endotracheal tube adapter allows rapid fiberoptic bronchoscopy-guided placement, thereby reducing ventilation interruptions during apneic oxygenation.

• A tailored lung-protective ventilation strategy using low peak inspiratory pressures makes major thoracic procedures—such as a right pneumonectomy for massive congenital cystic adenomatoid malformation complicated by hydrops fetalis—hemodynamically feasible and safe even in fragile neonates.

## Introduction

Congenital cystic adenomatoid malformation (CCAM) is a rare developmental anomaly of the lung characterized by the overgrowth of terminal bronchioles. CCAM is characterized by cystic structures that can lead to severe respiratory distress, mediastinal shift, and cardiovascular collapse in the neonatal period. In severe cases, these masses increase intrathoracic pressure, thereby impeding venous return and triggering the development of hydrops fetalis starting from the fetal period.^1, 2^

Indications for one-lung ventilation (OLV) in neonatal surgery include the resection of lesions such as CCAM, congenital lobar emphysema, and bronchopulmonary sequestration, or the management of intractable pulmonary hemorrhage.^1^ However, the application of OLV in neonates poses profound anatomical and physiological challenges. These primarily include the rapid onset of hypoxia within seconds—driven by a low functional residual capacity—and an elevated risk of mucosal edema due to a narrow airway caliber, compounded by the fact that adult double-lumen tubes (DLT) are disproportionately large for the neonatal trachea.^1, 3^ Since DLT cannot be utilized in the pediatric and infant age groups, fiberoptic bronchoscopy (FOB)-guided selective bronchial intubation or bronchial blocker (BB) applications are employed to achieve lung isolation in neonates.^3, 4^

The aim of this case report is to share our successful management of a neonatal patient undergoing pneumonectomy for CCAM-induced hydrops fetalis, in which OLV was effectively provided via FOB-guided left mainstem bronchial intubation.

## Case Presentation

A male neonate weighing 2500 g was delivered by emergency cesarean section at 30 weeks of gestation because of hydrops fetalis and a massive right-sided thoracic lesion diagnosed antenatally. Apgar scores were 4 and 7 at 1 and 5 minutes, respectively. The infant was intubated immediately after birth with a 2.5-mm uncuffed endotracheal tube and transferred to the neonatal intensive care unit. Initial arterial blood gas (ABG) analysis revealed severe respiratory acidosis (pH 6.96, PaCO_2_:108 mmHg, PaO_2_:56 mmHg). On postnatal day 3, progressive respiratory failure and hemodynamic instability prompted emergency surgical intervention.

Preoperative examination demonstrated generalized edema and ascites consistent with hydrops fetalis. Oxygen saturation was 89%, and heart rate was 105 beats/min. ABG analysis showed pH 7.20, PaCO_2_:61 mmHg, and PaO_2_:36 mmHg despite mechanical ventilation.

Written informed consent was obtained from the patient’s legal guardians prior to the procedure. Standard monitoring was applied in the operating room, and vascular access was obtained through an umbilical venous catheter. Induction and maintenance of anaesthesia were achieved with sevoflurane. Because commercially available DLT and BB are unsuitable for neonatal airways, a selective left mainstem intubation was planned.

To accommodate the FOB, the initial 2.5-mm uncuffed endotracheal tube was exchanged for a 3.5-mm cuffed endotracheal tube, the smallest tube diameter that allows internal passage of the pediatric bronchoscope. The cuffed tube was selected  to achieve complete lung isolation based on its structural fit, with the cuff intentionally left deflated throughout the procedure to prevent mucosal injury. The 3.5-mm tube was advanced into the left main bronchus under fiberoptic guidance. By removing the tube adaptor, the procedure offered a pragmatic, time-efficient alternative, thereby minimizing ventilation interruptions during apneic oxygenation. To minimize the risk of airway injury, the cuff remained completely deflated throughout the procedure, bronchoscopic guidance was used to avoid repeated airway manipulations, and the  tube position was carefully confirmed after patient positioning. No clinical evidence of airway injury was observed (Figure 1).

Pressure-controlled ventilation was maintained throughout the procedure using a lung-protective strategy. Ventilation parameters were adjusted to keep peak inspiratory pressures below 20 cm H_2_O with a positive end-expiratory pressure of 5 cm H_2_O and a respiratory rate of 35-40 breaths/min. A strategy of mild permissive hypercapnia was adopted, targeting an arterial PaCO_2_ below approximately 60 mmHg while maintaining an arterial pH above 7.25. The inspired oxygen fraction (FiO_2_) was adjusted according to continuous pulse oximetry and ABG analysis to maintain adequate oxygenation throughout OLV. During right pneumonectomy, transient desaturation episodes related to mediastinal manipulation were successfully managed by increasing inspired oxygen concentration and temporarily interrupting surgical traction. The procedure lasted approximately three hours and was completed without intraoperative complications; the non-functioning cystic lung tissue was successfully removed by pneumonectomy (Figure 2). The patient was transferred to the neonatal intensive care unit while intubated.

## Discussion

Neonatal OLV can theoretically be achieved using either BB or selective mainstem bronchial intubation. Although BB are widely utilized in thoracic surgeries in older pediatric patients, their application in extremely low birth weight neonates is severely constrained by technical difficulties with placement, unstable positioning, and the inherent risk of partial or complete airway obstruction. Furthermore, the small internal diameter of neonatal endotracheal tubes significantly restricts device manipulation, which increases procedural complexity and escalates the likelihood of ventilation interruption.^1, 3, 4^

Although upsizing the endotracheal tube in a premature neonate may increase the risk of airway complications, including mucosal ischemia, vocal cord injury, post-extubation edema, and subglottic stenosis, accurate fiberoptic-guided placement was considered essential to achieve reliable lung isolation during emergency pneumonectomy.^1, 5^ In the absence of appropriately sized BBs or DLT, selective mainstem intubation was the only technically feasible option for this patient’s airway.

Selective mainstem intubation using a single-lumen endotracheal tube is not without limitations, as excessive advancement may obstruct the upper lobe bronchus or result in inadequate lung isolation. However, fiberoptic bronchoscopic guidance allows precise tube placement beyond the carina while preserving upper lobe ventilation and facilitates immediate confirmation and repositioning after patient positioning or surgical manipulation.^3^ Consequently, this technique may represent a reliable strategy for lung isolation in extremely premature neonates, in whom BB placement is technically challenging. Therefore, the choice of lung-isolation technique should be individualized according to patient size, airway anatomy, available equipment, and surgical requirements.^1, 2, 4^

## Conclusion

This case suggests that selective left mainstem bronchial intubation may provide an effective means of achieving lung isolation in selected premature neonates undergoing thoracic surgery when BBs or other dedicated lung-isolation devices are impractical. FOB guidance facilitated accurate tube placement while allowing continued ventilation or apneic oxygenation during airway manipulation. These factors may have contributed to a shorter, more efficient lung-isolation procedure. Careful patient selection and individualized airway planning remain essential for the successful management of complex neonatal thoracic cases.

## Ethics

**Informed Consent:** Written informed consent was obtained from the patient’s legal guardians prior to the procedure.

## Figures and Tables

**Figure 1:**
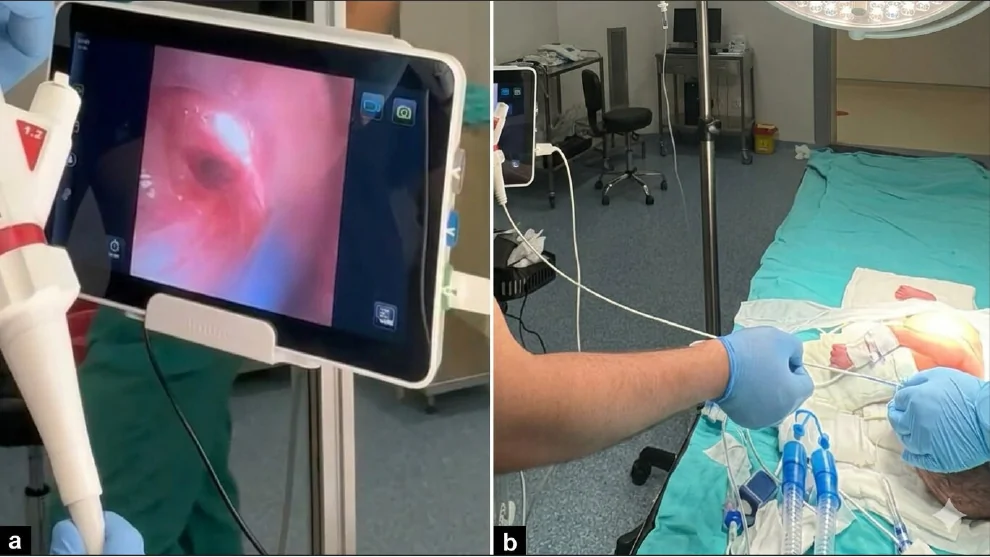
Fiberoptic-guided selective left mainstem bronchus intubation. a) Intraoperative bronchoscopic view confirming the correct positioning and patency within the left main bronchus. b) Clinical procedure in the operating room demonstrating the controlled advancement of the ultra-thin fiberoptic scope through the small-caliber endotracheal tube (with the tube adaptor removed) to achieve lung isolation.

**Figure 2:**
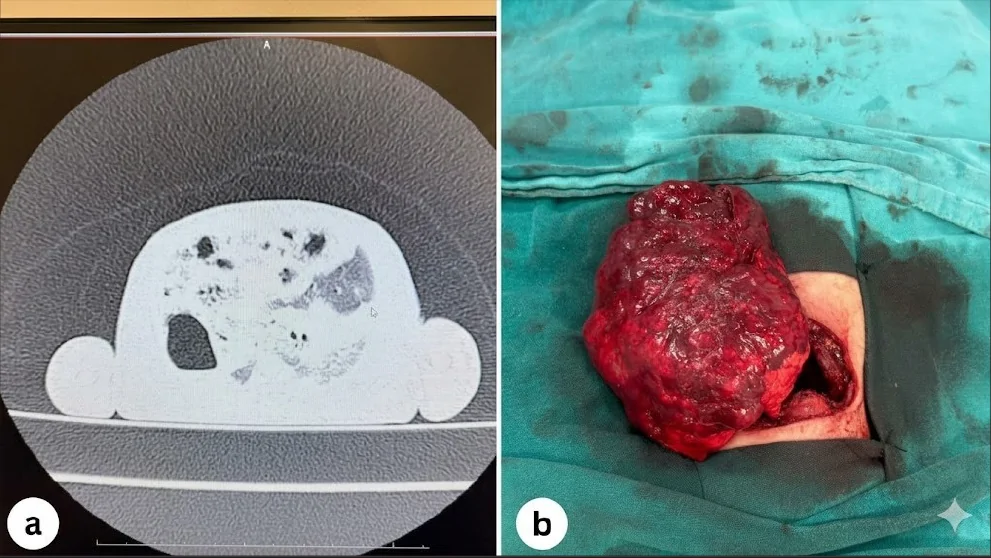
Preoperative imaging and macroscopic view of the resected specimen. a) Axial chest computed tomography scan demonstrating a massive right-sided congenital cystic adenomatoid malformation causing significant mediastinal shift, b) postoperative macroscopic view of the completely excised cystic right lung specimen following the formal pneumonectomy procedure.
